# Supplementary material for: miR-1285-3p targets TPI1 to regulate the glycolysis metabolism signaling pathway of Tibetan sheep Sertoli cells
Source: PLoS One. 2022 Sep 22;17(9):e0270364. doi: 10.1371/journal.pone.0270364 (PMC9499212; doi:10.1371/journal.pone.0270364)
Supplement: S1 Table — (DOCX) [file pone.0270364.s001.docx]

Table S1. Information of si-TPI1 sequence

| Name | Sequence | |
| --- | --- | --- |
|  | sense（5'-3'） | antisense（5'-3'） |
| si-TPI1-1 | CUGGCAUGAUCAAAGAUCUTT | AGAUCUUUGAUCAUGCCAGTT |
| si-TPI1-2 | CCAAGGUCAUCGCAGAUAATT | UUAUCUGCGAUGACCUUGGTT |
| si-TPI1-3 | CUGAGUUCGUCGACAUCAUTT | AUGAUGUCGACGAACUCAGTT |
