# Supplementary material for: miR-1285-3p targets TPI1 to regulate the glycolysis metabolism signaling pathway of Tibetan sheep Sertoli cells
Source: PLoS One. 2022 Sep 22;17(9):e0270364. doi: 10.1371/journal.pone.0270364 (PMC9499212; doi:10.1371/journal.pone.0270364)
Supplement: S2 Table — (DOCX) [file pone.0270364.s002.docx]

Table S2 Information of miRNA sequence

| **Name** | **Sense（5'-3'）** | **Antisense（5'-3'）** |
| --- | --- | --- |
| **miR-1285-3p mimics** | UCUGGGCAACAAAGUGAGACCU | GUCUCACUUUGUUGCCCAGAUU |
| **mimics NC** | UUCUCCGAACGUGUCACGUTT | ACGUGACACGUUCGGAGAATT |
| **miR-1285-3p inhibitor** | AGGUCUCACUUUGUUGCCCAGA |  |
| **inhibitor NC** | CAGUACUUUUGUGUAGUACAA |  |
