# Supplementary material for: miR-1285-3p targets TPI1 to regulate the glycolysis metabolism signaling pathway of Tibetan sheep Sertoli cells
Source: PLoS One. 2022 Sep 22;17(9):e0270364. doi: 10.1371/journal.pone.0270364 (PMC9499212; doi:10.1371/journal.pone.0270364)
Supplement: S3 Table — (DOCX) [file pone.0270364.s003.docx]

Table S2. Information of primer sequence

| **Gene** | **GenBank No.** | **Sequences (5’-3’)** | **Length/bp** |
| --- | --- | --- | --- |
| ***Bcl2*** | XM_012103831.3 | F: GGTGCCTATCTGGGCCATAA  R: CGTTGAGCCTGAAAGCTGTTTG | 195 |
| ***Bax*** | XM_027978592.1 | F: GCCCTTTTGCTTCAGGGTTT  R: TCGGAAAACATTTCAGCCGC | 235 |
| ***PCNA*** | XM_004014340.4 | F: TAGCCGTGTCATTGCGACTC  R: CGGTAAGTGTCGAAGCCCTC | 209 |
| ***caspase3*** | XM_015104559.2 | F: CTGCAACGTTGTGGCTGAAC  R: CCGGAGTCCACTGATTTGCT | 126 |
| ***ENO1*** | XM_015099234.2 | F: CTTCACCGCGAAAGGTCTCT  R: GGGCAGGCGCAATAGTTTTA | 158 |
| ***PKM*** | XM_004010279.4 | F: ATGATCAAGAAGCCTCGCCC  R: GTCGCTGGTAATGGGTGACA | 222 |
| ***LDHB*** | XM_027967824.1 | F: GGGCATCCAGAAGGACCTAA  R: AGACAAAAGCGAACTGTGCTC | 130 |
| ***LDHA*** | XM_027967935.1 | F: GGTTCCATTTAAGGCCCCTC  R: ACCTAAAGGAACCGGGAGTG | 125 |
| ***MCT1*** | XM-004006335 | F: TTAGCAATTATGGCAAGAGT  R: TACAAGTCCCATAGAAGGTC | 106 |
| ***GAPDH*** | NM_001190390.1 | F: TTATGACCACTGTCCACGCC  R: TCAGATCCACAACGGACACG | 216 |
| ***PGK*** | [NM_179576.3](https://www.ncbi.nlm.nih.gov/entrez/viewer.fcgi?db=nucleotide&id=1063693491) | F: TCTTGGGGTGTTGTCCAAGC  R: CGAACGTTACAGAGGCTGGT | 106 |
| ***PGAM1*** | XM_027960181 | F: TCCCAATTCCACATTAGCCAT | 173 |
|  |  | R: CTGCTCCTTAGTAATAAACCAC |  |
