# Supplementary material for: miR-1285-3p targets TPI1 to regulate the glycolysis metabolism signaling pathway of Tibetan sheep Sertoli cells
Source: PLoS One. 2022 Sep 22;17(9):e0270364. doi: 10.1371/journal.pone.0270364 (PMC9499212; doi:10.1371/journal.pone.0270364)
Supplement: S4 Table — (DOCX) [file pone.0270364.s004.docx]

Table S4. Information of primer sequence

| Primer name | Forward primer (5’-3’) | Reverse primer（5'-3'） |
| --- | --- | --- |
| miR-1285-3p | TCTGGGCAACAAAGTGAGACCT | Universal reverse |
| miR-3187-5p | CCTGGGCAGCGTGTGGCTGAAGG | Universal reverse |
| miR-133a-3p | TTGGTCCCCTTCAACCAGCTGT | Universal reverse |
| miR-6860 | ACTGGGCAGGGCTGTGGTGAGT | Universal reverse |
| miR-612-3p | GCTGGGCAGGGCTTCTGAGCTCCTT | Universal reverse |
| miR-6878 | CTGGCCTCTTCTTTCTCCTAG | Universal reverse |
| U6 | GGAACGATACAGAGAAGATTAGC | TGGAACGCTTCACGAATTTGCG |

Note: Universal reverse indicates the downstream universal primer by the kit (Mir-XTM miRNA First-Strand Synthesis Kit)
