# Supplementary material for: miR-1285-3p targets TPI1 to regulate the glycolysis metabolism signaling pathway of Tibetan sheep Sertoli cells
Source: PLoS One. 2022 Sep 22;17(9):e0270364. doi: 10.1371/journal.pone.0270364 (PMC9499212; doi:10.1371/journal.pone.0270364)
Supplement: Attachment — Submitted filename: Response to Reviewer.docx [file pone.0270364.s006.docx]

**Response to Reviewer 1 Comments**

We thank the reviewer for the time in closely viewing our manuscript. Most of the suggestions have been adopted in the revision.

Major comments:

**Point 1:** The abstract is poorly written.

**Response 1:** Thanks for your comments, we have revised the abstract section, and the specific revision results are as follows: Glycolysis in Sertoli cells (SCs) can provide energy substrates for the development of spermatogenic cells. Triose phosphate isomerase 1 (TPI1) is one of the key catalytic enzymes involved in glycolysis. However, the biological function of TPI1 in SCs and its role in glycolytic metabolic pathways are poorly understood. On the basis of a previous research, we isolated primary SCs from Tibetan sheep, and overexpressed *TPI1* gene to determine its effect on the proliferation, glycolysis, and apoptosis of SCs. Secondly, we investigated the relationship between *TPI1* and miR-1285-3p, and whether miR-1285-3p regulates the proliferation and apoptosis of SCs, and participates in glycolysis by targeting *TPI1*. Results showed that overexpression of *TPI1* increased the proliferation rate and decreased apoptosis of SCs. In addition, overexpression of *TPI1* altered glycolysis and metabolism signaling pathways and significantly increased amount of the final product lactic acid. Further analysis showed that miR-1285-3p inhibited *TPI1* by directly targeting its 3'untranslated region. Overexpression of miR-1285-3p suppressed the proliferation of SCs, and this effect was partially reversed by restoration of *TPI1* expression. In summary, this study shows that the miR-1285-3p/TPI1 axis regulates glycolysis in SCs. These findings add to our understanding on the regulation of spermatogenesis in sheep and other mammals.

**Point 2:** All the figure legends should be revised—for example, Fig2: Changing of TPI1 protein in SCs after silence or overexpression. What about the images or graphs in the figure? Don’t they need any explanation? What about the statistical significance? What methods were used? Fig 2 is cited at the end of the paragraph. Very hard to understand.

**Response 2:** Thanks for your comments, I have modified it in the text.

**Point 3:** In the text, it was mentioned si-PGAM1, whereas in the figure TPI1.

**Response 3:** Thanks for your comments, this is a mistake in my negligent text, it should be si-TPI1, I have made changes in the text.

**Point 4:** In the 3rd figure and 3.3, what is CCK8? How can first-time readers understand?

**Response 4:** Thanks for your comments, CCK8 is *Cell counting kit-8,* I have modified it in the text.

**Point 5:** Difference in overexpression or silence after 24 h of transection: What is transection? The number of cells in the si-PTPI1 group was significantly lower than that in the NC group. What is si-PTTPI1? Further qRT-PCR was used to detect the changes in the expression of proliferation, cycle and apoptosis-related genes at the mRNA level. What is cycle? The above typos are from a single paragraph. I haven’t included the English corrections! Similarly, every paragraph has typos and English corrections.

**Response 5:** Thanks for your comments, I have double checked the article and corrected the mistakes.

**Point 6:** Materials and methods (Isolation and culture of Sertoli cells): The authors are suggested to provide the culture purity data (GATA4/SOX9 immunostaining) and the expression of TPI1 (immunostaining, immunoblotting) in cultured Sc in the main figure. This is important as contamination by other testicular cell types (also known to express TPI1) needs to be ruled out.

**Response 6:** Thanks for your comments, I have modified the results section, and the specific modification results are as follows:

***3.1 Identification of Tibetan sheep primary SCs***

Immunofluorescence staining of SCs with specific antibody GATA4 and TPI1 antibody showed that purified SCs could synthesize and express SCs-specific binding protein GATA4, and staining with TPI1 antibody showed that TPI1 protein was expressed in SCs. The results could rule out contamination by other testis cell types, and the purified SCs could be used in subsequent experiments (Fig. 1).

**Fig. 1 Immunofluorescence staining of primary SCs of Tibetan sheep (20×).**

**Point 7:** For how many days were the cells cultured in vitro? After how many days were the cells transfected, and what was the transfection efficiency? The authors should provide information regarding the same in the material and methods section.

**Response 7:** Thanks for your comments, SCs were cultured in vitro and purified to the F5 generation for transfection, and the cells at 24, 48 and 72 after transfection were collected, and the transfection efficiency was detected by fluorescence qRT-PCR. It was found that the transfection efficiency at 48h was the highest. Therefore, other follow-up experiments were selected 48h after transfection. I have modified it in the article.

**Point 8:** The authors have studied the effect of TPI1 on Sc proliferation and apoptosis. There is a dogma that Sc proliferates during the neonatal infantile period and stops proliferating onset of puberty. Thus authors are suggested to check the expression of TPI1 changes during pubertal maturation of Sc. Along the same lines, does the expression of mi-1285-3p change during the functional maturation of Sc at puberty? Incorporation of this data would improve the quality of the manuscript.

**Response 8:** Thanks for your comments, our research group tested the expression of TPI1 in the testis of Tibetan sheep at different developmental stages, and found that the expression of its protein and mRNA before sexual maturity (3 mouth) was significantly lower than that of sexual maturity (1 year) and somatic maturity (3 years), and stabilized after sexual maturity.

**Point 9:** How does knock-down/over-expression of TPI1 regulate the mRNA levels of glycolytic pathway genes? Does TPI1 regulate the expression of these genes directly, or is the change in gene expression indirect (due to a shift in metabolic flux as a consequence of TPI1 knockdown/over-expression)? The authors should comment on this in the discussion section.

**Response 9:** Thanks for your comments, as one of the key enzymatic molecules in the glycolytic pathway, TPI1 catalyzes the conversion of dihydroxyacetone phosphate to glyceraldehyde-3-phosphate [11]. Then, under the action of GADPH, PGK, PGAM1, ENO, PKs, etc., it is converted into pyruvate and generates ATP, and then finally lactate is generated under the action of lactate dehydrogenase [9]. Therefore, changes in *TPI1* gene expression can directly change the changes in the downstream genes of the glycolytic pathway, and through these genes regulate the changes of enzymes in the glycolytic metabolic pathway, and finally change the content of its final product, lactate.

**Point 10:** The authors are suggested to provide separate figures for the TPI1 knockdown and over-expression experiments along with the consequent effect(s) of knockdown/over-expression of TPI1 on Sc function (proliferation, apoptosis, gene expression etc..).

**Response 10:** Thanks for your comments, the first half of the article is the test of *TPI1* gene knockdown or overexpression, and also explores the effect of *TPI1* gene knockdown or overexpression on SCs proliferation, apoptosis and glycolysis metabolic pathways.

**Point 11:** Which gene was used to normalize the gene expression data? This needs to be mentioned in the materials and methods section.

**Response 11:** Thanks for your comments, *β-actin* gene was used to normalize the gene expression data, I have added in the methods section of the text.
**Point 12:** Results section 3.4- the authors state that “the expression of ENO1, PKM, LDHA, LDHB, MCT1, GAPDH, and PGK genes were significantly down-regulated in the si-PGAM1 group (P<0.05), indicating the over-expression of the PGAM1 gene could increase the expression of downstream genes while silencing the PGAM1 gene could reduce the expression of downstream genes”. PGAM1 needs to be replaced with TPI1 in the text.

**Response 12:** Thanks for your comments, I have changed “PGAM1” to “TPI1”in the text.

**Point 13:** There are major grammatical errors and awkward sentences in the manuscript which need to be corrected.
**Response 13:** Thanks for your comments, I have found native English speakers to revise the grammar and sentences.

Minor:
**Point 1:** Line 2. Repetition of sentences. breeds in China; Tibetan sheep (Ovis aries) is one of the three major coarse-haired sheep breeds in China. breeds in China.

**Response 1:** Thanks for your comments, I have modified it in the text.

**Point 2:** Page no-8 In the initial introduction, the importance of spermatogenesis should be brief and crisp. It covers half of the introduction with repeated sense.

**Response 2:** Thanks for your comments, I have modified it in the text.

**Point 3:** The authors should provide details of Ab used for isolation, polyclonal or monoclonal?

**Response 3:** Thanks for your comments, the construction of the vector was completed by Genewiz Biological. We just verified the double-enzyme digestion of the overexpression vector synthesized by them, so the single-clonal and polyclonal information was not described in detail in the method.

**Point 4:** Would the authors mind providing precise details of the sample collection procedure (collected from a slaughterhouse or by sacrifice) and the number of testicles collected for each experiment?

**Response 4:** Thanks for your comments, three well-grown 3-month-old Tibetan sheep were selected and brought back to the laboratory for intravenous injection of sodium pentobarbital, no heartbeat, continuous involuntary breathing for 2-3 min, no blink reflex, and then bilateral testes tissues were collected. I have modified it in methods section.

**Point 5:** No need to rewrite the material method in the result section.

**Response 5:** Thanks for your comments, I have modified the results section.

**Point 6:** The author should provide good-quality images. Fig 5 and 6 captions are not readable.

**Response 6:** Thanks for your comments, I have modified the Fig 5 and 6.

**Response to Reviewer 2 Comments**

We thank the reviewer for the time in closely viewing our manuscript. Most of the suggestions have been adopted in the revision.

Major issues:
**Point 1:** It was not possible to confirm the existence of a binding site to miR-1285-3p in the TPI1 gene using miRanda, because the website is no longer available. However, using miRDB (http://mirdb.org) it is possible to estimate two binding sites between TPI1 and miR-1285-3p in 3’ UTR, in humans. However, according to this database, there are 41 miRNAs targeting the TPI1 gene, and the miR-1285-3p is not the miRNA with higher “score”. The authors state in the introduction that this miRNA was selected because it is the most expressed in the Tibetan Sheep among the miRNAs with high affinity for the TPI1 gene. Yet, miR-1285-3p has 895 targets and TPI1 is not within the top 50 targets. Among the miR-1285-3p targets with higher score than TPI1 are several genes that could be involved in cell proliferation and metabolic regulation. Therefore, it is unlikely that the action of miR-1285-3p in Sertoli Cells is restricted to the regulation of TPI1 gene. To claim that, the authors would have to characterise the transcriptome of the Sertoli Cells transfected with the TPI1 inhibitor.

**Response 1:** Thanks for your comments, in our earlier studies, we explored the proteomics of sheep testis tissue and found that the TPI1 protein was expressed in sheep testis tissue. After conducting further functional annotations, we found that the TPI1 protein participates in the glycolytic metabolism pathway. In order to further verify its role in testicular development of Tibetan sheep, we further explored the expression and localization of TPI1 in testis tissue of Tibetan sheep at different developmental stages (prepubertal, sexual maturity and adulthood), and found that its mRNA and protein expression varied with the It increases with age and is mainly located in SCs. However, its role in SCs and its regulation of glycolytic metabolism have not been reported yet. This study mainly explored the role of TPI1 in the glycolysis process of Tibetan sheep SCs on the basis of previous studies. Since most of the previous research on TPI1 was in cancer, some reports on the regulation of miRNA on TPI1 were found in the literature when reviewing the literature, so combined with previous reports and prediction software (miRDB(http://www.mirdb.org) and Targetscan (http://www .targetscan.org), screened out several miRNAs that have a targeting relationship with TPI1, and found that miR-1285-3p had the highest expression in Sertoli cells, and the dual luciferase reporter gene assay was used to detect the high expression of miR-1285-3p. The targeting relationship between the three miRNAs and TPI1 was verified, and it was found that only miR-1285-3p had a targeting relationship, so this study directly selected miR-1285-3p for research.
**Point 2:** Related to the previous issue, the authors do not refer the parameters to predict miRNAs targeting TPI1 using miRanda, especially which animal database was used. Although the primers were specific to Tibetan Sheep, the target prediction was likely performed using a database for another species. This limitation must be discussed.

**Response 2:** Thanks for your comments, in this study, the first prediction was based on the human database through miRDB. After predicting that miR-1285-3p has a targeting relationship with *TPI1*, the sequence of sheep miR-1285-3p was downloaded through miRNAbase. The seed region of miR-1285-3p was aligned with the 3'UTR region of the ovine *TPI1* gene, and it was found that it was completely complementary. Therefore, it was shown that *TPI1* also has a targeting relationship with miR-1285-3p in sheep, so follow-up experiments were carried out.
**Point 3:** It is not clear to me how the miR-1285-3p mimic is expressed in Sertoli Cells by means of a plasmid, and the supplementary files are not available. How can the authors guarantee that the sequence will mimic the behaviour of the original miR-1285-3p? If the mimic sequence is expressed along the luciferase gene, then it must be processed post-transcriptionally. This must also be discussed by the authors.

**Response 3:** Thanks for your comments, the mimics and inhibitors are designed according to the gene sequence on the miRNAbase. The sense strand of the mimics sequence is the miRNA sequence, and the antisense strand is the sequence of the sense strand after removing the reverse complement of the last two bases and adding UU.The inhibitor sequence is the sequence after the complete reverse complementation of the base sequence. Synthetic miRNA mimics are chemical compounds of more than 20 bases, which simulate endogenous gene sequences through transfection with transfection reagents and then perform gene expression or knockdown.

**Point 4:** In section 3.5 and Figure 5, the authors mention the miR-3614-5p. Did the authors mean miR-1285-3p?

**Response 4:** Thanks for your comments, I made a typo, it should be miR-1285-3p, I have made changes in the text.

Minor issues:
**Point 1:** Please replace the term “testicles” by the term “testes” (singular “testis”).

**Response 1:** Thanks for your comments, I have modified it in the article.

**Point 2:** QRT-PCR -> qRT-PCR

**Response 2:** Thanks for your comments, I have modified it in the article.

**Point 3:** Multipanel figures must be labelled uniformly. In some figures the panels are ordered from left to right, top to bottom, but in other figures the authors label from top to bottom and only then from left to right. Although I understand the authors have a lot of information to show, please consider relegating some figure panels to supplementary data to improve the visualization of main manuscript figures.

**Response 3:** Thanks for your comments, I have modified the figures in the article.
