# Supplementary material for: miR-1285-3p targets TPI1 to regulate the glycolysis metabolism signaling pathway of Tibetan sheep Sertoli cells
Source: PLoS One. 2022 Sep 22;17(9):e0270364. doi: 10.1371/journal.pone.0270364 (PMC9499212; doi:10.1371/journal.pone.0270364)
Supplement: Attachment — Submitted filename: Response to Reviewer.docx [file pone.0270364.s007.docx]

**Response to Reviewer Comments**

We thank the reviewer for the time in closely viewing our manuscript. Most of the suggestions have been adopted in the revision.

**Point 1:** Particularly, it is important to state in the "Methods" the parameters of the miRNA target prediction tool, especially the species database. For instance, miRDB does not have a sheep database, so I guess the authors have relied on another database. Then, this issue must be discussed as a limitation of the study.

**Response 1:** Thanks for your comments, in this study, the first prediction was based on the human database through miRDB. It was found that 6 miRNAs had a targeting relationship with TPI1. Then the miRNA sequences of sheep were downloaded by miRNAbase software, and the sequences were compared, and it was found that their seed regions were the same. Then primers were designed to detect the expression of these 6 miRNAs in Tibetan sheep SCs. It was found that the expression level of miR-1285-3p was the highest in Tibetan sheep, and then the targeting relationship between miR-1285-3p and TPI1 was further predicted. It was found that the seed region of miR-1285-3p and the 3'UTR of TPI1 gene could be completely complementary. Therefore, it showed that miR-1285-3p had a targeting relationship with TPI1, and we further verified the targeting relationship with the double luciferase reporter gene.

**Point 2:** Also, I cannot find in the text any reference about the author's previous work, notably the claim that miR-1285-3p is expressed the most in testis of Tibetan sheep. This previous work is important to support the rationale for the present study, especially due to the lack of information and databases in the species.

**Response 2:** Thanks for your comments, the result that miR-1285-3p was most expressed in Tibetan sheep SCs was originally included in Figure S1 as supplementary information. According to expert advice, I have put this part of the results in 3.5 and put the picture in Fig. 6F. As shown below:

We studied the expression of 6 miRNAs predicted to be targeted to TPI1 in SCs and found that the expression level of miR-1285-3p in SCs was significantly higher than that of other miRNAs(*P*<0.05) (Fig. 6F).

**Fig. 6 TPI1 expression is downregulated by miR-1285-3p directly targeting of the 3’-UTR of TPI1.** A, TPI1 was found for the potential regulatory targets of miR-1285-3p using prediction tool. B, Dual-luciferase reporter assay analysis of the target relationship of miR-1285-3p with TPI1. F, Expression of miRNA in SCs. The TPI1 expression levels were determined by real-time PCR analysis (D) western blot (H) after transfection with the miR-1285-3p mimics or negative control or after transfection with the miR-1285-3p inhibitor or negative control in SCs. CCK-8 (E), Flow cytometry (G) and the expression of proliferation, cycle and apoptosis related genes (C) was used to detect the apoptosis of SCs after transfection miR-1285-3p mimics, negative control, miR-1285-3p inhibitor or negative control. The expression levels of downstream genes (J) and the content of glycolytic metabolic pathway products, energy and key enzymes (I) of SCs after transfection miR-1285-3p mimics, negative control, miR-1285-3p inhibitor or negative control.

**Point 3:** There is a erroneous reference to Figure 5 in line 335.

**Response 3:** Thanks for your comments, I have modified it in the text.
